# A Proteomic Approach for the Diagnosis of Bacterial Meningitis

**DOI:** 10.1371/journal.pone.0010079

**Published:** 2010-04-08

**Authors:** Sarah Jesse, Petra Steinacker, Stefan Lehnert, Martin Sdzuj, Lukas Cepek, Hayrettin Tumani, Olaf Jahn, Holger Schmidt, Markus Otto

**Affiliations:** 1 Department of Neurology, University of Ulm, Ulm, Germany; 2 Department of Neurology, University of Goettingen, Goettingen, Germany; 3 DFG Research Center for Molecular Physiology of the Brain, Goettingen, Germany; 4 Proteomics Group, Max-Planck-Institute for Experimental Medicine, Goettingen, Germany; University of California Merced, United States of America

## Abstract

**Background:**

The discrimination of bacterial meningitis (BM) versus viral meningitis (VM) shapes up as a problem, when laboratory data are not equivocal, in particular, when Gram stain is negative.

**Methodology/Principal Findings:**

With the aim to determine reliable marker for bacterial or viral meningitis, we subjected cerebrospinal fluid (CSF) to a quantitative proteomic screening. By using a recently established 2D-DIGE protocol which was adapted to the individual CSF flow, we compared a small set of patients with proven BM and VM. Thereby, we identified six potential biomarkers out of which Prostaglandin-H2 D-isomerase was already described in BM, showing proof of concept. In the subsequent validation phase on a more comprehensive collective of 80 patients, we could validate that in BM high levels of glial fibrillary acidic protein (GFAP) and low levels of soluble amyloid precursor protein alpha/beta (sAPPα/β) are present as possible binding partner of Fibulin-1.

**Conclusions/Significance:**

We conclude that our CSF flow-adapted 2D-DIGE protocol is valid especially in comparing samples with high differences in total protein and suppose that GFAP and sAPPα/β have a high potential as additional diagnostic markers for differentiation of BM from VM. In the clinical setting, this might lead to an improved early diagnosis and to an individual therapy.

## Introduction

Patients with meningitis do not always display typical clinical signs or characteristic laboratory parameters at the time of admission, even when the bacterial origin could be proven later on [1], [2]. Meningism is often missing especially in elderly patients [1] and young children [3]. Typical laboratory parameters in patients with bacterial meningitis are elevated CSF-leukocytes ≥1000/µl [4], CSF-protein ≥1 g/l [5], and CSF-lactate >3.0 mmol/l [6]. In blood samples, increase of leukocytes and of C-reactive protein (CRP, usually ≥100 µg/ml) can be found [7].

Nevertheless, patients at an early stage of the disease or after antimicrobial pre-treatment often show normal or inconclusive routine parameters [8], [9], [10], [11], so that further laboratory parameters to differentiate the meningitis would be beneficial. Actual, a mere pragmatic therapeutical proceeding is applied: every patient under the strong suspicion of meningitis is treated with a triple therapy of antiinfectious agents to cover as much pathogens as possible. This is a practical approach, but there are several reasons to improve the early treatment regime: firstly, there are adverse reactions that are underestimated especially in elderly patients and those with reduced renal function, leading to clinical complications other than meningitis-associated with the consequence of an extended hospitalisation. In the second place, pathogen-resistance against frequent and blindfold applied antiinfectives is a serious problem particularly with regard to the next ten or twenty years with the high risk of forfeiting therapeutic options. For these reasons, economic and specific application of pharmaceuticals is the basis for best efficient therapy–however, a precedent condition for this approach is the precise diagnosis.

The aim of our study was the identification of additional supportive proteins for the differentiation of meningitis, particularly with regard to those cases that are not clearly to be diagnosed at the first presentation. To minimize the inclusion of misdiagnosed patients, we deliberately concentrated here on cases with proven meningitis to find proteins that are typically involved in the pathophysiology of the disease. Recently we could establish a protocol especially for CSF proteomics, taking care to the fact that brain derived proteins in CSF are independent of total CSF-protein [12].

As methodical approach, we used fluorescent dye labelling, compatible with protein isoelectric focussing (2D-DIGE). The 2D-DIGE nowadays has the potential power to separate several thousand proteins on a single gel and has become a method of choice for quantitative proteomics [13], [14]. On that basis, we used this approach for the diagnosis of BM, with a special emphasis on differentiation from VM. We identified six promising marker-proteins out of more than 2500 spots. With the routinely established protein-biochemical methods ELISA and Westernblotting, these markers were then validated in a larger patient cohort.

## Results

### Patient data

For summary of all patient data see Table 1.

**Table 1:** Illustration of cardinal patient data.

group	n	age	m/f	leukocyte count/µl	Lactate (mmol/l)	Q_Alb_ (×10^3^)	total protein (mg/l)
BM 1	7	49±12	4/3	8078±1082	17.1±9.0	1673±3946	3606±1668
BM 2	21	55±14	13/8	4251±6018	10.4±7.1	703±2262	3816±4658
VM	25	53±21	15/10	72±92	1.9±0.4	10±5	753±335
CON	27	37±20	19/8	<5	<2.0	<8	<450

Table 1 indicates age, CSF-leukocyte count, CSF-lactate, Q_Alb_ and total protein that are declarated as mean±SD.

Abbreviations: Q_Alb_  =  CSF/serum albumin quotient; CSF  =  cerebrospinal fluid; BM1  =  bacterial meningitis of center 1; BM 2  =  bacterial meningitis center 2; VM  =  viral meningitis; CON  =  controls; m/f  =  male/female.

In the meningitis group, *Streptococcus pneumoniae* was identified in 15 patients, *Neisseria meningitidis* in three times. Five patients had *Listeria monocytogenes* and in five patients *Staphylococci aurei* were isolated.

### Identification of potential biomarkers for BM

**Figure 1:**
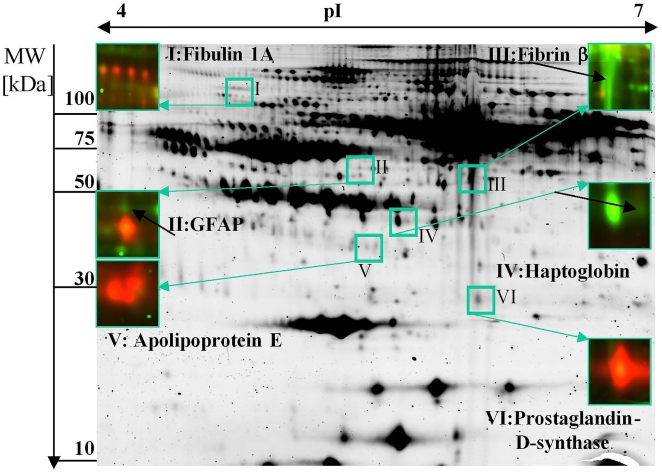
2D-DIGE - Illustration of the spots of interest. The 2D-DIGE gel of CyDye-labelled CSF proteins demonstrates four BM patients and four VM patients. The figure illustrates the identified proteins, numbered from I to VI. BM samples are demonstrated by the green-colored spots and are labelled with Cy 3; VM samples are demonstrated by the red-colored spots and are labelled with Cy 5. The red spot below GFAP is PEDF that in the sum of all gels is not regulated.

**Figure 2:**
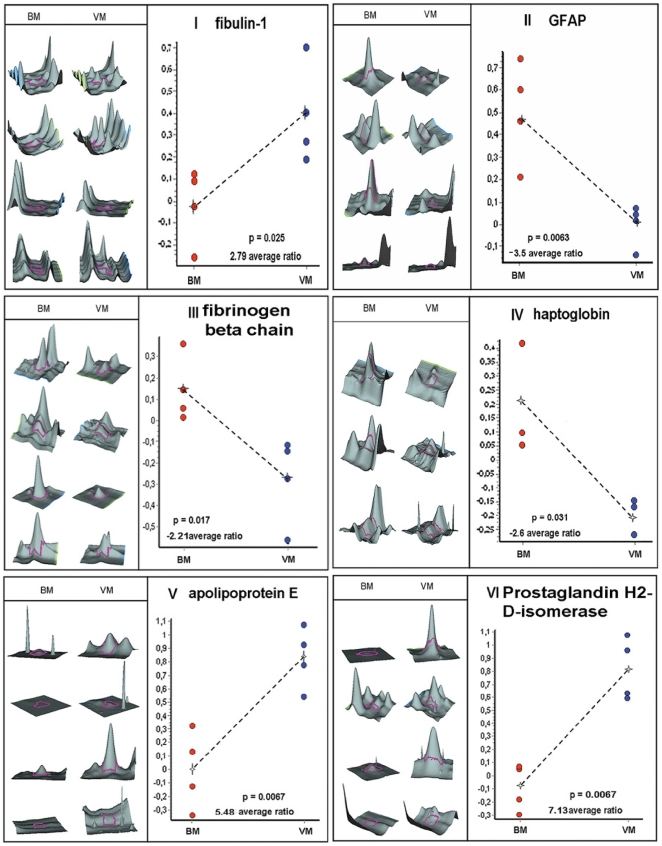
2D-DIGE - Detailed information of the proteins of interest. 2D-DIGE analyses with pixel volume distribution of the proteins I–VI, shown in figure 1 from numbers 1–4 BM, numbers 1–4 VM and average volume increased ratio. Using the DeCyder BVA Software, a Student's t-test applied to the four paired samples yielded a p-value within the 95^th^ percentile confidence level.

In the 2D-DIGE approach we obtained more than 2500 spots. Searching for spots which had a significant higher spot volume (at least 2.0 times different) and a p-value below 0.05, we received 10 candidates matching this criterion. After staining with colloidal coomassie and mass spectrometric protein identification based on peptide mass and sequence information obtained by MALDI-ToF-MS, six out of 10 candidate proteins were identified (Figure 1). Figure 2 and Table 2 as well as Figure S3 and Table S1 illustrate data of spot identification.

**Table 2:** 2D-DIGE analyses and identification of selected CSF proteins.

Spot No.	p value t-test	Ratio BM/VM	Protein name	Swiss-Prot accession	MW [kDa]	pI
I	0.025	2.79	Fibulin-1	P23142	81.3	5.1
II	0.0063	3.50	Glial fibrillary acidic protein	P14136	49.9	5.4
III	0.017	−2.21	Fibrinogen beta chain	P02675	56.6	8.5
IV	0.031	−2.60	Haptoglobin	P00738	45.9	6.1
V	0.006	5.48	Apolipoprotein E	P02649	36.2	5.7
VI	0.006	7.13	Prostaglandin-H2 D-isomerase	P41222	21.2	7.7

Table 2 illustrates cardinal data, including Swiss Prot accession number, MW and pI.

Abbreviations: BM/VM  =  bacterial/viral meningitis; MW  =  molecular weight; pI  =  isoelectric point.

### Preclinical-validation of proteins relevant for differential diagnosis

#### Prostaglandin-H2 D-isomerase and Haptoglobin

As the Prostaglandin-H2 D-isomerase (or prostaglandin-D-synthase/β-trace) was already described in the differential diagnosis of BM [15], [16], we refrained from validating this protein. Concerning Haptoglobin, others found a diagnostic relevance within the 1^st^ and 14^th^ day of disease, so that we did not follow-up this protein [17].

#### Fibulin-1, Fibrinogen beta chain and Apolipoprotein E

For Fibulin-1, Fibrinogen beta chain and Apolipoprotein E, immunoblotting was done as validation step (data not shown). For Fibrinogen beta chain (Figure S1), we detected elevated levels in BM in comparison to CON (p = 0.005) but not a difference between BM versus VM (p = 0.131).

For Fibulin-1 as well as Apolipoprotein E, no difference could be seen in the 1D immunoblots. Assuming different isoforms of these proteins, we performed 2D immunoblots for Fibulin-1 as well as Apolipoprotein E and found in the case of Fibulin-1 a wide distribution of isoforms (Figure S2), which were suggestive for different isoform-allocation, but difficult to be differentiated by 2D-immunoblot.

#### Fibulin-1, sAPPα and sAPPβ

**Figure 3:**
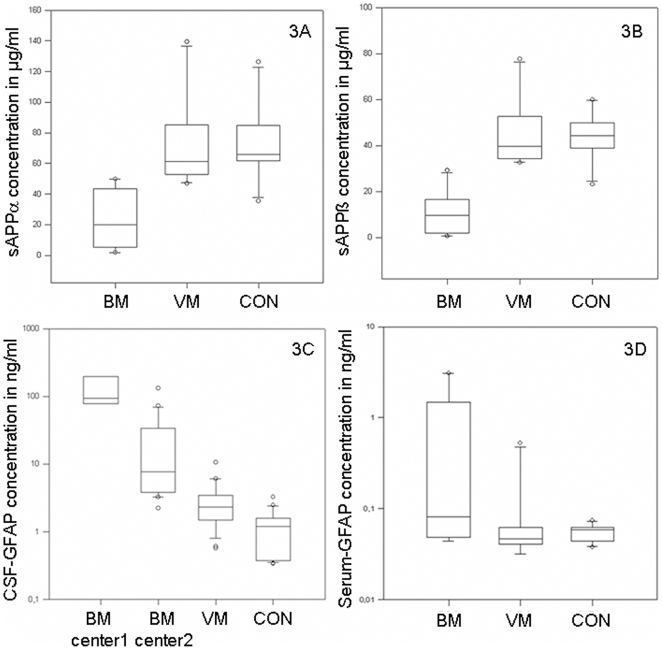
GFAP and sAPPα/β - Data of the ELISA and Multiplex. Boxplots of sAPPα (3A) as well as sAPPβ levels (3B) in CSF of patients with BM, VM and CON are indicated. Part 3C and 3D demonstrates GFAP-levels in CSF (3C) and serum (3D). In CSF, the comparison between center one and center two is indicated for BM. Plot shows 10th, 25th, 50th, 75th, and 90th percentiles and outliers.

Because of the above mentioned different isoforms of Fibulin-1 in the 2D immunoblot, we investigated binding proteins of fibulin-1 like APP-processing products [18], [19]. In a pilot experiment, we investigated sAPPα/β in a small cohort of patients (10 BM, 10 VM and 10 CON) using multiplex ELISA [20]. Here, significantly decreased sAPPα/β values for BM in comparison to VM and CON were detected (p = 0.001) (Table 3 and Figure 3A/B).

**Table 3:** Multiplex data of sAPPα/β.

group	n	sAPPα in µg/ml as mean±SD	sAPPβ in µg/ml as mean±SD
BM	10	22.81±19.45; [±13.92]	10.51±9.43; [±6.75]
VM	10	73.03±29.71; [±21.25]	45.31±14.83; [±10.61]
CON	10	73.01±23.99; [±17.16]	43.94±10.24; [±7.33]

Data of multiplex enzyme linked immunosorbent assay for sAPPα as well as sAPPβ in BM, VM and CON, comprising 10 patients per group. The 95% CI for each group is indicated in brackets.

Abbreviations: sAPPα/β  =  soluble amyloid beta precursor protein.

#### GFAP ELISA of CSF and serum samples

For GFAP, a commercially available ELISA was performed, identifying a significant increase in patients with BM (p<0.001) in comparison to VM and CON. As GFAP levels in BM were out-of range of the standard curve, dilutions of up to 1∶50 had to be applied.

As the CSF-GFAP values in BM patients differed in both centers (Ulm 7 patients and Göttingen 21 patients), separate analyses of the data were performed. In VM (27 patients) as well as CON (25 patients), no differences between the centers were found. In serum, we could not detect different values of GFAP in a pilot experiment (10 patients per group, p = 0.068, Figure 3C/D and Table 4).

**Table 4:** ELISA of GFAP in BM and VM meningitis.

group	GFAP in CSF in ng/ml; mean±SD	GFAP in serum in ng/ml; mean±SD
BM 1	176.96±195.30; [±180.63]	0.75±1.23; [±0.882]
BM 2	22.53±32.37; [±15.15]	-
VM	2.94±2.24; [±0.944]	0.10±0.15; [±0.109]
CON	1.15±0.77; [±0.305]	0.06±0.01; [±0.008]

Because of distinct GFAP values in both centers (Ulm and Göttingen), we performed separate analyses for the BM group. In CSF, 7 BM patients of center 1 (BM 1), 21 BM patients of center 2 (BM 2), 25 VM patients as well as 27 CON patients were examined. In serum, ELISA was performed with 10 patients per group. The 95% CI for each group is indicated in brackets.

### Correlations

To test correlations between GFAP and standard parameters of CSF, we calculated Spearman correlations that are listed in Table 5.

**Table 5:** Correlation of GFAP to routine CSF data.

tested parameter	group	r	p
GFAP vs CSF-lactate	BM	0.296	0.179
	VM	-0.064	0.765
GFAP vs Q_Alb_	BM	0.075	0.736
	VM	-0.022	0.917
GFAP vs CSF-total protein	BM	0.266	0.228
	VM	0.172	0.417
GFAP vs CSF-leukocyte count	BM	0.136	0.541
	VM	-0.418	0.042
GFAP vs sAPPα	BM	-0.750	0.038
	VM	0.750	0.038
GFAP vs sAPPβ	BM	-0.679	0.074
	VM	0.536	0.181

Illustration of Spearman correlation coefficient (r) with the respective p-values (p). To test possible correlations between GFAP and standard parameters, we performed Spearman correlations and found a negative/positive correlation between GFAP and sAPPα in BM/VM.

## Discussion

In the differential diagnosis of early stages of BM, routine laboratory results can be misleading [21]. CSF-leukocyte counts can be lower than 100/µl [22] heralding a severe clinical course, the CSF-leukocyte differentiation is also not an accurate tool: when the leukocyte count is below 1000/µl, more than 30% of patients show a CSF-lymphocytosis [23]. CSF-lactate concentration seems to be more reliable [24], here a cut-off value of 3.0 mmol/l was proposed by Berg and co-workers [6]. Nevertheless, two of their patients in rather early stages showed lactate concentrations below this cut-off. Although the additional measurement of serum CRP appears to be a good marker for the differentiation of BM versus VM [25], there still is a diagnostic uncertainty [7], [26].

Regarding biomarker approaches, two main hypothetical factors for analysis can be opted: firstly, the investigation of proteins specifically related to microbial pathogenicity like secreted/surface or immunogenetic proteins, as often performed by protein-biochemical methods. Here, multiplex PCR with primer sequences to detect both, bacterial [27], [28] as well as viral DNA [29] represents a suitable method in cases with either clinically suspected bacterial or viral origin of the CNS infection. The second basis for biomarker research is the identification of proteins representing the host-specific response to a damaging agent–usually performed as proteomic approach and better applicable in those cases where the underlying pathogenic agent is not to be anticipated.

We therefore decided on a proteomic approach to search for additional biomarkers. As methodical technique, we used our CSF-optimised, volume-based 2D-DIGE [12], a recently published approach that takes the physiological CSF-flow of proteins into consideration. Roughly 70% of lumbar CSF used for diagnostic approaches consists of blood-derived proteins especially albumin and immunoglobulins. However, the composition of ventricular CSF is quite different. The amount of blood derived proteins is lower so that proteins feature a concentration gradient from the ventricular space towards the lumbar part, increasing in the case of blood-proteins and decreasing in the case of intracerebral-proteins [30], [31], [32]. Even after depletion of the major high abundand proteins, lots of proteins are still blood-derived, so that an artificial normalisation to total protein amount in CSF can lead to wrong assumptions traced back to the mainly detected blood-derived proteins [12]. This still hold true after albumin and immunoglobulin depletion. Our just established approach should be preferred for CSF proteomics, especially for inflammatory diseases and neurodegenerative disorders where a reduced CSF-flow can be observed with the consequence of high total-protein levels of which BM is an extreme-model.

Using this 2D-DIGE, we detected six relevant proteins for the differential diagnosis of BM versus VM. For this proteome step, only few patient samples were included being a critical point of the study but our volume based proteome-method represents a proof-of concept confirmed by the fact that we did not identify blood-derived proteins. By standarization towards total protein amount, we would have missed GFAP as diagnostic marker, as in BM higher total protein amounts can be observed. However for the initial proteomic approach, very few patients were used so that we possibly have missed some markers which were different in BM compared to VM, but did not match our strict criteria we used for later validation. Also in this respect, BM is a model disease as a clear differentiation between BM and VM is necessary and an overlap even is some patients would be a reason not to follow- up such a candidate marker. Having in mind that we used for our initial proteomic screen only some clear well defined patients, the following validation step is even more important. This was done in our study, especially as we even used samples from two independent centers to avoid the measurement of a center-effect. A more comprehensive patient collective in the subsequent preclinical validation phase was obligate, so that we examined 80 persons by protein-biochemical methods. Here, we decided on the basis of the spot volume data to pursue the examination of GFAP with regard to its differentially diagnostic potential. Using ELISA, highest levels could be seen in BM that could - in a pilot experiment - not be reproduced in serum.

Interestingly, we found different concentrations of GFAP in both centers with higher levels in center one. For VM and CON, these variations of protein concentration did not appear. To exclude at least - as a possible reason for this difference - distinct pre-treatment protocols, we performed thaw/freezing cycles of CSF and found a tendency of GFAP-decreased concentrations, indicating the importance of preanalysis for research and the need to take this into account by interpretation of the study-results. However, in center two the time between storage and freezing was up-to one week that could emerge as a possible cause for lower GFAP in the samples.

To estimate the clinical importance of GFAP, we compared its values with the routine standard parameters and found no correlations in the BM group and a barely correlation between GFAP and leukocyte count for VM. Because of this diagnostic independence, we suppose that the measurement of GFAP has a potential as additional marker for the diagnosis of BM.

The failure to validate proteins like Fibulin-1 as well as Apolipoprotein E in the 1D immunoblot might be caused by the proteomic approach itself, the available detection antibodies and the fact that we might not detect the special isoform which was seen in the proteomic approach. On the basis of the 2D immunoblot data showing a different isoform pattern of Fibulin-1, we assumed a pathophysiological relevance of this protein in meningitis. Nevertheless, the results imply that not only this protein but also other proteins known to co-function with or be regulated by Fibulin-1 are changed with respect to CSF expression levels. Since Fibulin-1 is known to be involved in APP regulation, we had a closer look to this protein [19]. For sAPPα/β, we found significantly decreased levels in BM, possibly explaining the low levels of Aβ1-42 in CSF of BM which were described by others [33] as a phenomenon of less or downregulated APP that is available for further processing. In BM, we could also see a significant negative correlation of GFAP and sAPPα, so that a combined analytical investigation of these proteins might be of differential diagnostic relevance. Regarding APP in patients with AD, Blennow et al. found no different levels of sAPPα/β in comparison to controls suggesting no change in APP expression in sporadic AD [34]. Mattsson et al. investigated the activity of the β-site APP cleaving enzyme (BACE1) in CSF of patients with multiple sclerosis. BACE1 cleaves APP which results in the release of N-terminal β-cleaved sAPPβ. They found reduced levels of sAPPα/β compared to controls, but the reductions in sAPP products seem not to be specific to MS because of similar results seen in Lupus erythematodes as an example of another autoimmune disease [35].

Regarding the biological relevance as well as the pathophysiological aspects of GFAP in meningitis, a probable astrogliosis followed by bacterially triggered inflammation of the CNS could be the morphological correlate of the observed GFAP release into the CSF. It is not yet clear if this astroglia-proliferation is due to bacterial toxins or to overwhelming activation of early immune responses [36].

Best investigations of astrogliosis were performed in chronic multiple sclerosis and in mouse models of experimental autoimmune encephalomyelitis (EAE) [23]. Here, an overexpression of GFAP was detected that is discussed to be essential for normal white matter architecture and its absence is said to cause late-onset CNS dysmyelination [37]. Moreover, recent data based on the EAE-model indicate increased GFAP-levels being a promising marker of the chronic disease stage in generally and of the secondary progressive disease phase in particular in patients with multiple sclerosis [38]. Similar investigations concerning disease conversion from clinically mild stages to more severe forms showed antigen detection of measles, rubella and varicella zoster (the so called MRZ-reaction) having prognostic relevance to predict conversion to MS in patients with a clinically isolated syndrom [39].

Apart from these data, a discriminative aspect of GFAP in BM and VM has not yet been evaluated. Moreover, we were able to demonstrate a proof-of-concept for this protein concerning proteomic approaches with subsequent validation by protein-biochemical approaches.

As GFAP is possibly less dependent on antibiotic treatment in the early phase of CNS infection than CSF-lactate and CSF-leukocyte count, its measurement might be useful in situations where patients have been pre-treated with antimicrobial agents. Additionally, the examination of sAPPα as downregulated protein may be of further relevance in the differential diagnosis of meningitis, maybe reflecting pre-synaptical pathological [40] or protective processes [18] as seen in neurodegenerative diseases like AD [41], [42]. Further studies with the aim to validate the role of APP–best with robust tests for sAPPα/β–in the clinical workflow will investigate if this protein is a complementary marker in those cases where GFAP levels are not pathbraking. Additional investigations with an inhomogeneous group of patients in a routine setting should be performed to evaluate the diagnostic relevance and the reproducibility of these biomarkers.

## Materials and Methods

### Ethics Statement

This study was conducted according to the principles expressed in the Declaration of Helsinki. Taking of the CSF and blood samples from humans were conducted with the approval of the local ethics committee (Ethik-Kommission der Medizinischen Fakultät der Universitäten Ulm und Göttingen, approval numbers: 8801 and 100305). All patients provided written informed consent for the collection of samples and subsequent analysis.

### Subject source, Sample pre-treatment and Patients

To discover potential biomarkers, we investigated CSF of 8 patients (4 BM, 4 VM, sample bank: University hospital Ulm) in the proteomic approach. For the validation phase, a larger collective comprising up to 80 patients, prospectively collected between 2007/2008 (University hospitals in Ulm [center one] and Göttingen [center two]) was used (28 BM, 25 VM and 27 CON).

After routine analysis of native CSF, supernatants were aliquoted and stored at −80°C in Ulm within two hours. In Göttingen, CSF was kept at 4°C up to one week before final storage at −80°C. Samples were collected and aliquoted in polypropylene cups. Patients with intracerebral or subarachnoidal haemorrhage, chronic inflammatory diseases like multiple sclerosis, autoimmune diseases, seizures without signs of CNS-infection, symptoms traced back to psychiatric indispositions or meningitis signs of undefined etiology were excluded. For BM, only patients with positive microbiological verification (either by blood culture, CSF culture, Gram stain, PCR or agglutination tests) were included [43], [44]. The diagnosis of VM was performed by the leukocyte count, the usual cell-staining, the lactate concentration as well as the specific identification of HSV1 that was the causing agent in 3 cases. A positive PCR on the first days of infection or an antibody index above at least 1.6 after one to two weeks is a precondition to assume an infection with this viral agent.

### CyDye Labelling

Proteomic analysis via 2D-DIGE was done with a volume-based normalization as described previously [12]. CSF samples were albumin- and immunoglobulin-depleted (depletion kit, GE Healthcare) according to the manufacturer's instructions with the exception, that 750 µl of the slurry was used per 1 ml original volume of CSF. For CSF proteome comparison, four CSF samples of each group were compared by the mixed internal standard methodology described by Alban [45]. Each sample (400 µl CSF, approximately 80–120 µg) was labelled with 200 pmol of Cy3 and Cy5. The single samples were labelled either Cy3 or Cy5 for a dye-switched comparison to avoid potential dye-to-protein preferences. The mixed internal standard contains aliquots of each individual sample (corresponding to 100 µl CSF) and was labelled with Cy2 in the same dye-to-CSF ratio. The samples were combined and diluted 1.33x by a stock solution for subsequent IEF.

### 2D Gel Electrophoresis and Imaging

IEF was also done as described previously [12]. Second dimension SDS-PAGE was performed with homogeneous 12.5% gels (254×200 mm) according to Tastet et al. [14] at 3.5 W/gel overnight at 20°C. The fluorescence signals of the Cy-labelled protein samples were imaged using a scanner recording emission wavelengths of 520 nm (Cy2), 580 nm (Cy3) and 670 nm (Cy5) (DIGE-Imager, GE Healthcare). Proteins were post-stained with colloidal coomassie. Spots of interest were excised manually and subjected to mass spectrometric protein identification.

### In-gel digest and mass spectrometry

Manually excised gel plugs were subjected to an automated platform for the identification of gel-separated proteins [46]. Using an Ultraflex MALDI-ToF mass spectrometer (Bruker Daltonics), a peptide mass fingerprint (PMF) and six fragment ion spectra for each sample were recorded automatically under the control of the FlexControl 3.0 operation software (Bruker Daltonics). PMF spectra were calibrated externally on the basis of nine-point near-neighbor calibrant spectra (Peptide Calibration Standard II, Bruker Daltonics). Annotation of monoisotopic peptide signals in the m/z range 800–4000 by the SNAP algorithm (S/N threshold = 4, quality factor threshold = 50, number of peaks limited to 100) and generation of the corresponding peak lists was performed with the FlexAnalysis 3.0 post-processing software (Bruker Daltonics). Background signals corresponding to typical trypsin autolysis peptides and to a CHCA cluster at m/z 833 were removed from the peak list. Fragment ion spectra were post-processed as above with slightly different settings for the SNAP-based peak detection (S/N threshold = 3, quality factor threshold = 30, number of peaks limited to 200).

### Database search

PMF and MS/MS data sets were batch-processed using the BioTools 3.1 software (Bruker Daltonics) as interface to the Mascot 2.2 software (Matrix Science) licensed in-house. Database searches were performed in the Swiss-Prot primary sequence database Sprot 56.9 (release of 03-Mar-2009, 412525 entries), restricted to the taxonomy *homo sapiens* (20402 entries). Carboxamidomethylation of Cys was specified as fixed and oxidation of Met as variable modification. One trypsin missed cleavage was allowed. Mass tolerances were set to 100 ppm for PMF searches and to 100 ppm (precursor ions) and 0.7 Da (fragment ions) for MS/MS ion searches. The minimal requirement for accepting a protein as identified was at least one peptide sequence match above identity threshold in coincidence with at least 20% sequence coverage assigned in the PMF.

### Westernblotting

Westernblotting was done according to standard protocols [12]. SDS-PAGE with 12% polyacrylamide gels was performed according to Laemmli [47]. After first dimension, equilibration and electrophoresis, proteins were transferred onto PVDF membrane (Schleicher & Schuell, Germany) in semi dry blot equipment [48]. The first antibodies were diluted 1∶20000 (mAb Apolipoprotein E, Epitomics #1930-1) and 1∶1000 (mAb Fibrinogen beta chain, American Diagnostics #ADI350) as well as 1∶500 (mAb Fibulin-1, Santa Cruz #sc-55470) in PBS, 0.0075% Tween-20, containing 5% dry milk powder (Roth, Germany).

As secondary antibodies, peroxidase-conjugated goat anti-rabbit Ig (Dianova, Germany, 1∶2000, #111-035-003) or goat-anti mouse (Dako, Germany, 1∶2000, #P0447) were applied and proteins were visualized by chemiluminescence according to the manufacturer's instructions (ECL plus, GE Healthcare).

### Human GFAP ELISA

Human GFAP was detected via ELISA (BioVendor, Germany). The assay was performed according to manufacturer's instructions. The analytical limit of detection was 0.033 ng/ml [49].

### Multiplex enzyme-linked immunosorbent assay

Multiplex ELISA for sAPPα/β was performed according to manufacturers instructions (Meso Scale Discovery, USA). Concentrations were calculated from standard curves fitted by four parameter equation using the supplied analysis software (mesoscale). The analytical limit of detection was 120 pg/ml for sAPPα and 52 pg/ml for sAPPβ. For detailed instructions of this multiplex ELISA see reference [50].

### Statistical analysis

Band volumes (adjusted for membrane background) of the immunoblots were determined using the Quantity One software (BioRad, Germany). The comparison of protein concentrations between the different subgroups was based on calculations using Kruskal-Wallis test. Correlations were performed by Spearman correlation. Calculations and comparisons were performed by the software SigmaStat 3.5 (Systat Software Inc, USA).

## Supporting Information

Figure S1 Westernblot-analysis of Fibrinogen beta chain. Band volumes of fibrinogen beta chain in 1D immunoblot are indicated, adjusted to membrane background.
(0.07 MB DOC)

Figure S2 2D-Westernblot of Fibulin-1. Illustration of 2D immunoblots with Fibulin-1 antibody in BM (left) as well as VM (right). The Westernblots show different isoform patterns of this protein in the meningitis samples.
(0.97 MB DOC)

Figure S3 MS-spectra
(0.21 MB PDF)

Table S1 Additional data of protein identification. Columns 4–6 refer to PMF searches; columns 7–9 refer to MS/MS ion searches.
(0.05 MB DOC)

## References

[1] Rasmussen HH, Sorensen HT, Moller-Petersen J, Mortensen FV, Nielsen B (1992). Bacterial meningitis in elderly patients: clinical picture and course.. Age Ageing.

[2] van de Beek D, de Gans J, Spanjaard L, Weisfelt M, Reitsma JB (2004). Clinical features and prognostic factors in adults with bacterial meningitis.. N Engl J Med.

[3] Pena JA, Jimenez L (1999). [Prognosis of bacterial meningitis].. Rev Neurol.

[4] Karandanis D, Shulman JA (1976). Recent survey of infectious meningitis in adults: review of laboratory findings in bacterial, tuberculous, and aseptic meningitis.. South Med J.

[5] Deisenhammer F, Bartos A, Egg R, Gilhus NE, Giovannoni G (2006). Guidelines on routine cerebrospinal fluid analysis. Report from an EFNS task force.. Eur J Neurol.

[6] Berg B, Gardsell P, Skansberg P (1982). Cerebrospinal fluid lactate in the diagnosis of meningitis. Diagnostic value compared to standard biochemical methods.. Scand J Infect Dis.

[7] Hansson LO, Axelsson G, Linne T, Aurelius E, Lindquist L (1993). Serum C-reactive protein in the differential diagnosis of acute meningitis.. Scand J Infect Dis.

[8] Coll MT, Uriz MS, Pineda V, Fontanals D, Bella F (1994). Meningococcal meningitis with ‘normal’ cerebrospinal fluid.. J Infect.

[9] Onorato IM, Wormser GP, Nicholas P (1980). ‘Normal’ CSF in bacterial meningitis.. JAMA.

[10] Polk DB, Steele RW (1987). Bacterial meningitis presenting with normal cerebrospinal fluid.. Pediatr Infect Dis J.

[11] Heckenberg SG, de Gans J, Brouwer MC, Weisfelt M, Piet JR (2008). Clinical features, outcome, and meningococcal genotype in 258 adults with meningococcal meningitis: a prospective cohort study.. Medicine (Baltimore).

[12] Brechlin P, Jahn O, Steinacker P, Cepek L, Kratzin H (2008). Cerebrospinal fluid-optimized two-dimensional difference gel electrophoresis (2-D DIGE) facilitates the differential diagnosis of Creutzfeldt-Jakob disease.. Proteomics.

[13] Hu Y, Malone JP, Fagan AM, Townsend RR, Holtzman DM (2005). Comparative proteomic analysis of intra- and interindividual variation in human cerebrospinal fluid.. Mol Cell Proteomics.

[14] Tastet C, Lescuyer P, Diemer H, Luche S, van Dorsselaer A (2003). A versatile electrophoresis system for the analysis of high- and low-molecular-weight proteins.. Electrophoresis.

[15] Tumani H, Nau R, Felgenhauer K (1998). Beta-trace protein in cerebrospinal fluid: a blood-CSF barrier-related evaluation in neurological diseases.. Ann Neurol.

[16] Tumani H, Reiber H, Nau R, Prange HW, Kauffmann K (1998). Beta-trace protein concentration in cerebrospinal fluid is decreased in patients with bacterial meningitis.. Neurosci Lett.

[17] Noris-Garcia E, Dorta-Contreras AJ, Gonzalez-Hernandez M, Bu Coifu-Fanego R, Padron-Gutierrez D (2008). [Clinical relevance of haptoglobin/IgG index and Boyer's score to the differential diagnosis of bacterial and viral meningitis].. Rev Neurol.

[18] Storey E, Cappai R (1999). The amyloid precursor protein of Alzheimer's disease and the Abeta peptide.. Neuropathol Appl Neurobiol.

[19] Ohsawa I, Takamura C, Kohsaka S (2001). Fibulin-1 binds the amino-terminal head of beta-amyloid precursor protein and modulates its physiological function.. J Neurochem.

[20] Lewczuk P, Hornegger J, Zimmermann R, Otto M, Wiltfang J (2008). Neurochemical dementia diagnostics: assays in CSF and blood.. Eur Arch Psychiatry Clin Neurosci.

[21] Ray P, Badarou-Acossi G, Viallon A, Boutoille D, Arthaud M (2007). Accuracy of the cerebrospinal fluid results to differentiate bacterial from non bacterial meningitis, in case of negative gram-stained smear.. Am J Emerg Med.

[22] Felgenhauer K, Kober D (1985). Apurulent bacterial meningitis (compartmental leucopenia in purulent meningitis).. J Neurol.

[23] Powers WJ (1985). Cerebrospinal fluid lymphocytosis in acute bacterial meningitis.. Am J Med.

[24] Cunha BA (2006). Distinguishing bacterial from viral meningitis: the critical importance of the CSF lactic acid levels.. Intensive Care Med.

[25] Tankhiwale SS, Jagtap PM, Khadse RK, Jalgaonkar SV (2001). Bacteriological study of pyogenic meningitis with special reference to C-reactive protein.. Indian J Med Microbiol.

[26] Paradowski M, Lobos M, Kuydowicz J, Krakowiak M, Kubasiewicz-Ujma B (1995). Acute phase proteins in serum and cerebrospinal fluid in the course of bacterial meningitis.. Clin Biochem.

[27] Boving MK, Pedersen LN, Moller JK (2009). Eight-plex PCR and liquid-array detection of bacterial and viral pathogens in cerebrospinal fluid from patients with suspected meningitis.. J Clin Microbiol.

[28] Chakrabarti P, Das BK, Kapil A (2009). Application of 16S rDNA based seminested PCR for diagnosis of acute bacterial meningitis.. Indian J Med Res.

[29] Del Prete R, Di Taranto AM, Lipsi MR, Natalicchio MI, Antonetti R (2009). Simultaneous detection of viruses and Toxoplasma gondii in cerebrospinal fluid specimens by multiplex polymerase chain reaction-based reverse hybridization assay.. New Microbiol.

[30] Reiber H (2001). Dynamics of brain-derived proteins in cerebrospinal fluid.. Clin Chim Acta.

[31] Reiber H (2003). Proteins in cerebrospinal fluid and blood: barriers, CSF flow rate and source-related dynamics.. Restor Neurol Neurosci.

[32] Otto M, Lewczuk P, Wiltfang J (2008). Neurochemical approaches of cerebrospinal fluid diagnostics in neurodegenerative diseases.. Methods.

[33] Sjogren M, Gisslen M, Vanmechelen E, Blennow K (2001). Low cerebrospinal fluid beta-amyloid 42 in patients with acute bacterial meningitis and normalization after treatment.. Neurosci Lett.

[34] Olsson A, Hoglund K, Sjogren M, Andreasen N, Minthon L (2003). Measurement of alpha- and beta-secretase cleaved amyloid precursor protein in cerebrospinal fluid from Alzheimer patients.. Exp Neurol.

[35] Mattsson N, Axelsson M, Haghighi S, Malmestrom C, Wu G (2009). Reduced cerebrospinal fluid BACE1 activity in multiple sclerosis.. Mult Scler.

[36] Weber JR, Tuomanen EI (2007). Cellular damage in bacterial meningitis: an interplay of bacterial and host driven toxicity.. J Neuroimmunol.

[37] Zhu H, Dahlstrom A (2007). Glial fibrillary acidic protein-expressing cells in the neurogenic regions in normal and injured adult brains.. J Neurosci Res.

[38] Linker RA, Brechlin P, Jesse S, Steinacker P, Lee DH (2009). Proteome profiling in murine models of multiple sclerosis: identification of stage specific markers and culprits for tissue damage.. PLoS One.

[39] Brettschneider J, Tumani H, Kiechle U, Muche R, Richards G (2009). IgG antibodies against measles, rubella, and varicella zoster virus predict conversion to multiple sclerosis in clinically isolated syndrome.. PLoS One.

[40] Lue LF, Kuo YM, Roher AE, Brachova L, Shen Y (1999). Soluble amyloid beta peptide concentration as a predictor of synaptic change in Alzheimer's disease.. Am J Pathol.

[41] Naylor R, Hill AF, Barnham KJ (2008). Is covalently crosslinked Abeta responsible for synaptotoxicity in Alzheimer's disease?. Curr Alzheimer Res.

[42] Rao KS, Hegde ML, Anitha S, Musicco M, Zucca FA (2006). Amyloid beta and neuromelanin–toxic or protective molecules? The cellular context makes the difference.. Prog Neurobiol.

[43] Van de Beek D, de Gans J, Spanjaard L (2004a). Clinical features and prognostic factors in adults with bacterial meningitis.. N Engl J Med.

[44] MiQ K, E, Dörries R, Geiß HK, Matz B, Neumann-Häfelin D, Pfister HW, Prange H, Schlüter D, Spellerberg B, Spencker FB (2001). Qualitätsstandards in der mikrobiologisch-infektiologischen Diagnostik..

[45] Alban A, David SO, Bjorkesten L, Andersson C, Sloge E (2003). A novel experimental design for comparative two-dimensional gel analysis: two-dimensional difference gel electrophoresis incorporating a pooled internal standard.. Proteomics.

[46] Jahn O, Hesse D, Reinelt M, Kratzin HD (2006). Technical innovations for the automated identification of gel-separated proteins by MALDI-TOF mass spectrometry.. Anal Bioanal Chem.

[47] Laemmli UK (1970). Cleavage of structural proteins during the assembly of the head of bacteriophage T4.. Nature.

[48] Wiltfang J, Smirnov A, Schnierstein B, Kelemen G, Matthies U (1997). Improved electrophoretic separation and immunoblotting of beta-amyloid (A beta) peptides 1–40, 1–42, and 1–43.. Electrophoresis.

[49] Jesse S, Steinacker P, Cepek L, von Arnim CA, Tumani H (2009). Glial fibrillary acidic protein and protein S-100B: different concentration pattern of glial proteins in cerebrospinal fluid of patients with Alzheimer's disease and Creutzfeldt-Jakob disease.. J Alzheimers Dis.

[50] Steinacker P, Hendrich C, Sperfeld AD, Jesse S, Lehnert S (2009). Concentrations of beta-amyloid precursor protein processing products in cerebrospinal fluid of patients with amyotrophic lateral sclerosis and frontotemporal lobar degeneration.. J Neural Transm.

