# Supplementary material for: A Proteomic Approach for the Diagnosis of Bacterial Meningitis
Source: PLoS One. 2010 Apr 8;5(4):e10079. doi: 10.1371/journal.pone.0010079 (PMC2851643; doi:10.1371/journal.pone.0010079)
Supplement: Figure S1 — Westernblot-analysis of Fibrinogen beta chain. Band volumes of fibrinogen beta chain in 1D immunoblot are indicated, adjusted to membrane background. (0.07 MB DOC) [file pone.0010079.s001.doc]

**
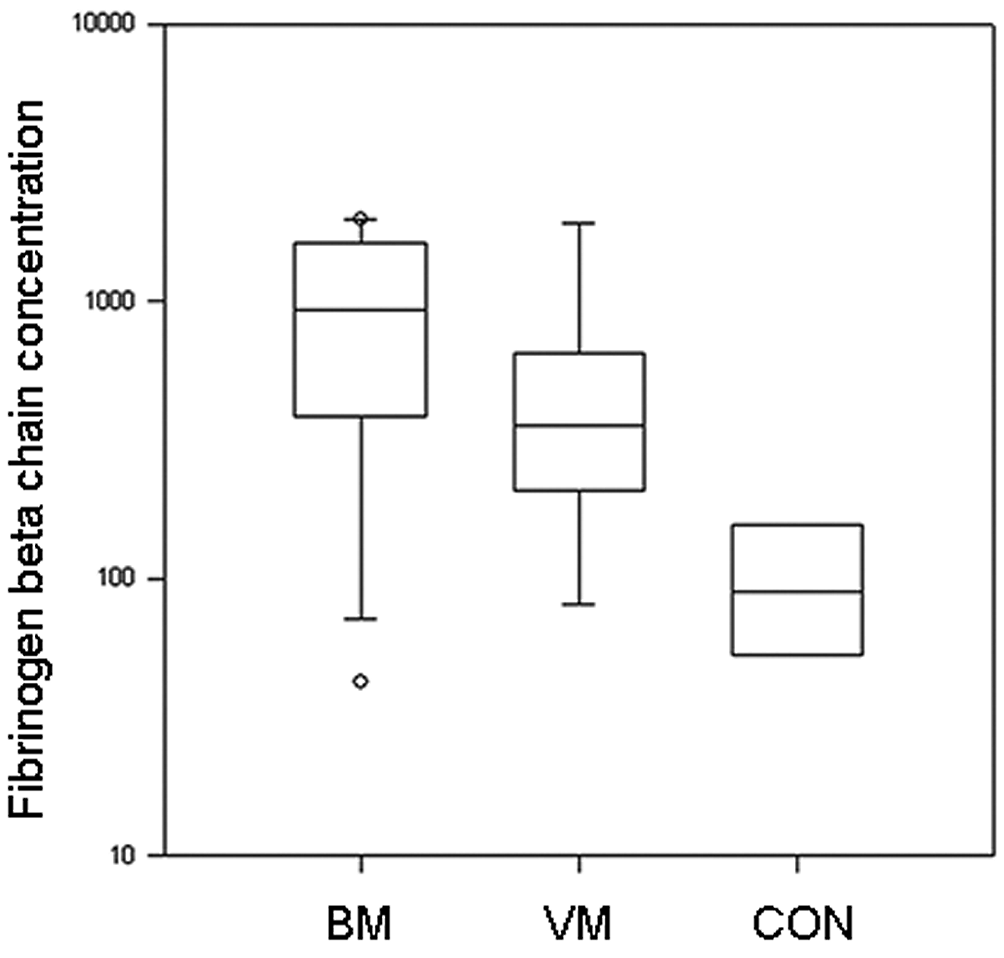
**

**Figure S1:** **Westernblot-analysis of Fibrinogen beta chain.**

Band volumes of fibrinogen beta chain in 1D immunoblot are indicated, adjusted to membrane background.
