# Supplementary material for: A Proteomic Approach for the Diagnosis of Bacterial Meningitis
Source: PLoS One. 2010 Apr 8;5(4):e10079. doi: 10.1371/journal.pone.0010079 (PMC2851643; doi:10.1371/journal.pone.0010079)
Supplement: Figure S2 — 2D-Westernblot of Fibulin-1. Illustration of 2D immunoblots with Fibulin-1 antibody in BM (left) as well as VM (right). The Westernblots show different isoform patterns of this protein in the meningitis samples. (0.97 MB DOC) [file pone.0010079.s002.doc]

**
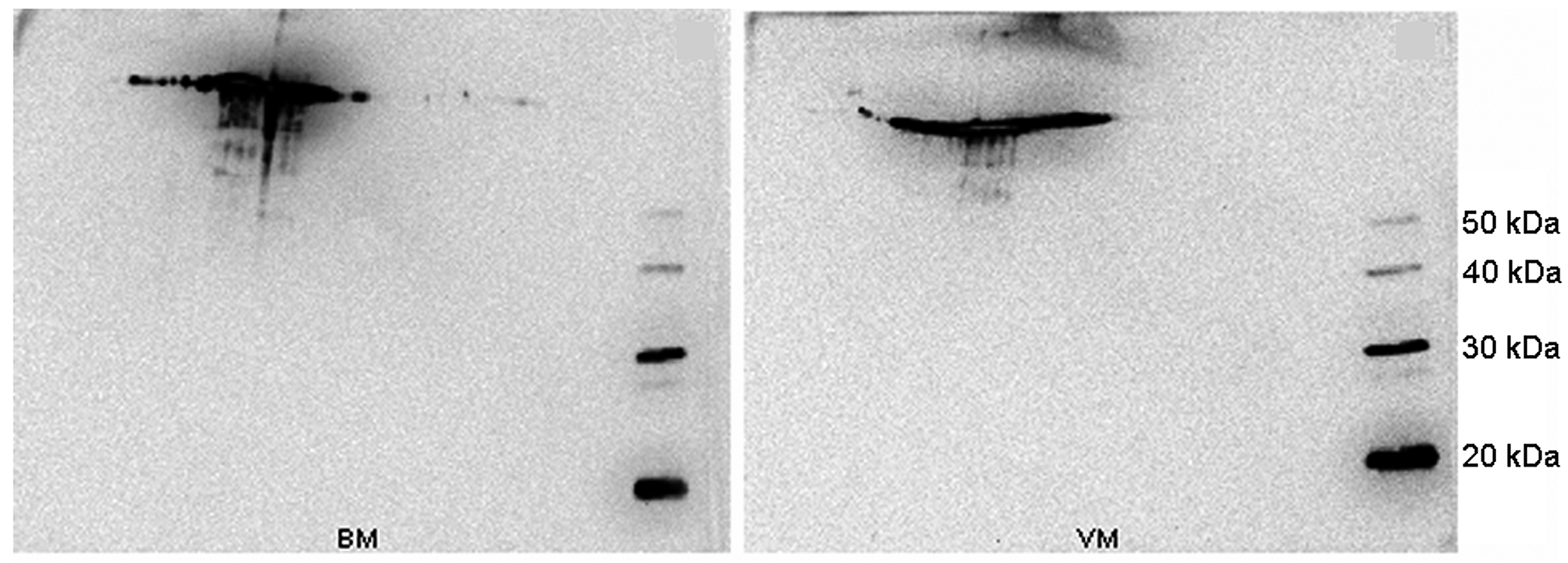
**

**Figure S2:** **2D-Westernblot of Fibulin-1.**

Illustration of 2D immunoblots with Fibulin-1 antibody in BM (left) as well as VM (right). The Westernblots show different isoform patterns of this protein in the meningitis samples.
