# Supplementary material for: A Proteomic Approach for the Diagnosis of Bacterial Meningitis
Source: PLoS One. 2010 Apr 8;5(4):e10079. doi: 10.1371/journal.pone.0010079 (PMC2851643; doi:10.1371/journal.pone.0010079)

Spot I: Fibulin-1 (P23142)
PMF score = 82

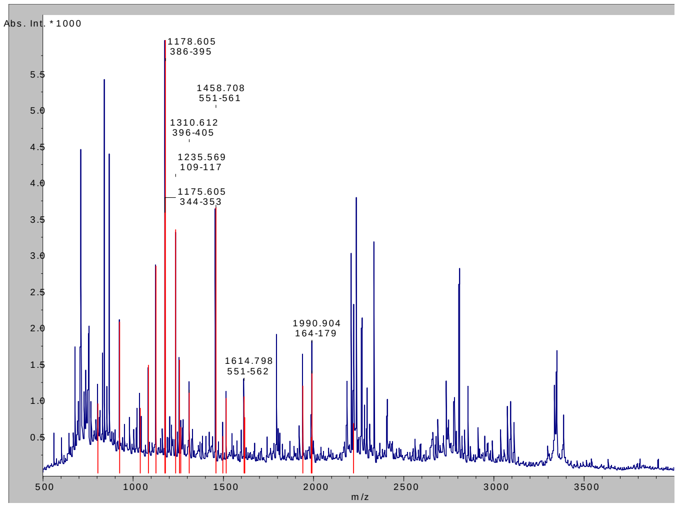

CCHCCLLGR, MS/MS ion score = 29

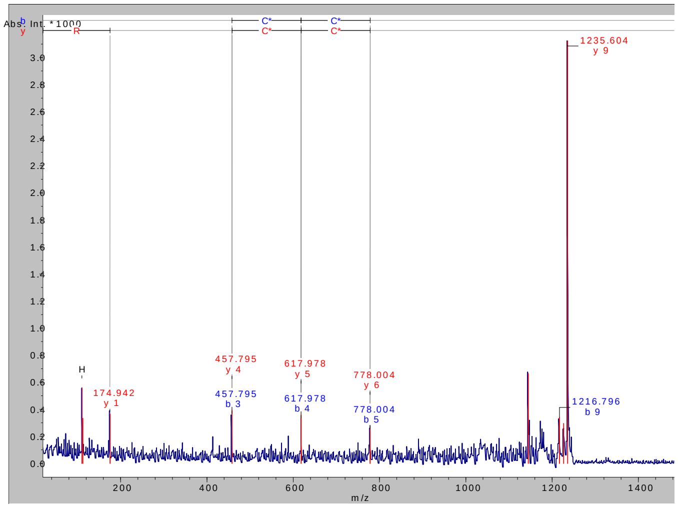

TGYYFDGISR, MS/MS ion score = 12

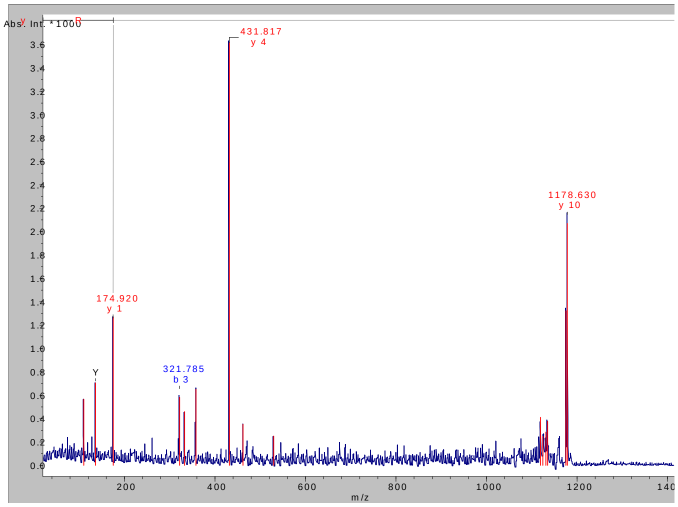

Spot II: Glial fibrillary acidic protein (P14136)

PMF score = 184

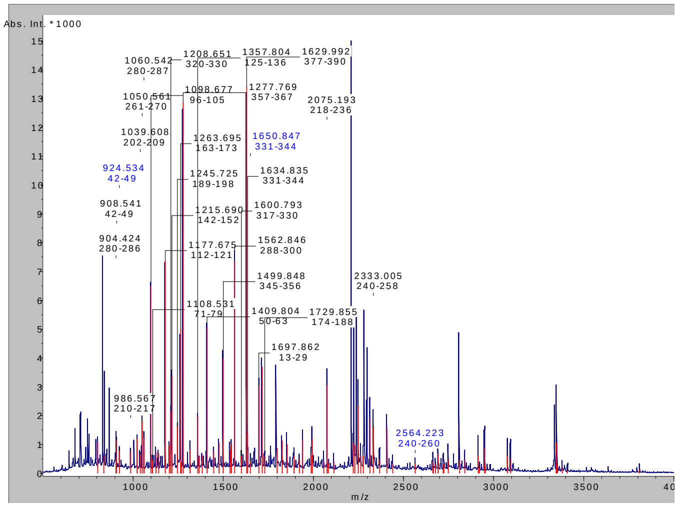

LADVYQAELR, MS/MS ion score = 59

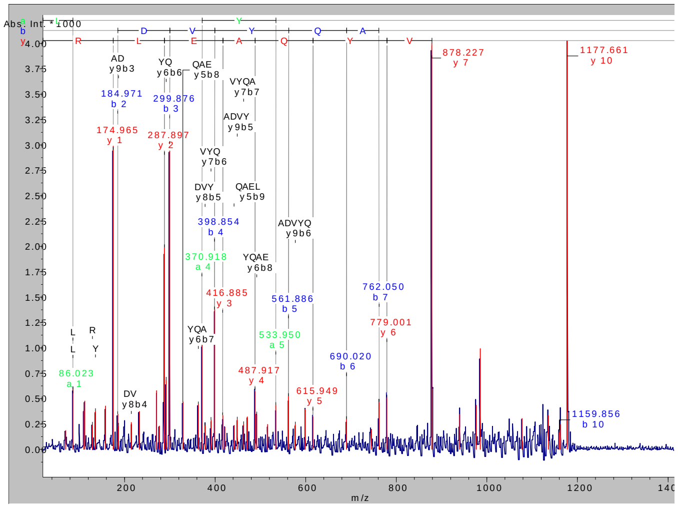

QLQSLTCDLESLR, MS/MS ion score = 54

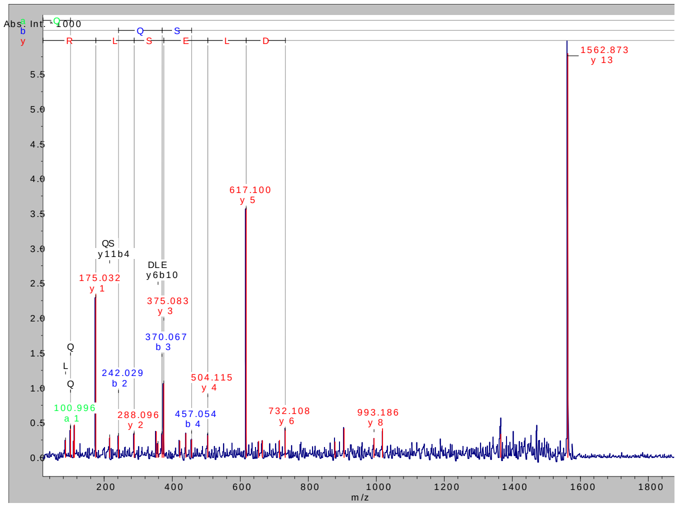

ITIPVQTFSNLQIR, MS/MS ion score = 48

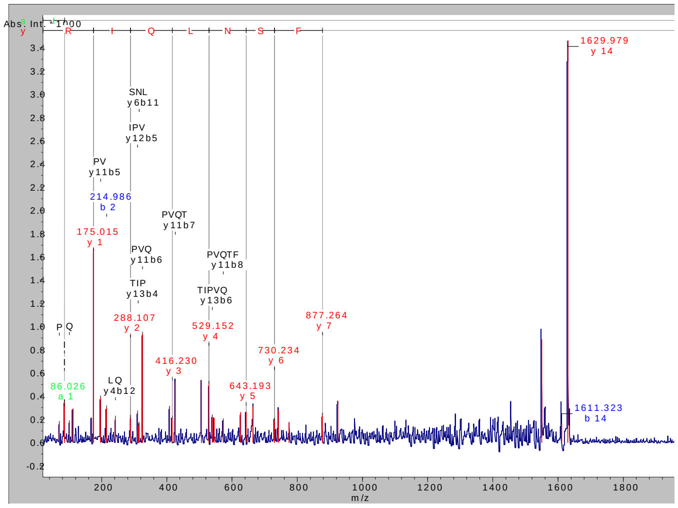

Spot III: Fibrinogen beta chain (P02675)
PMF score = 170

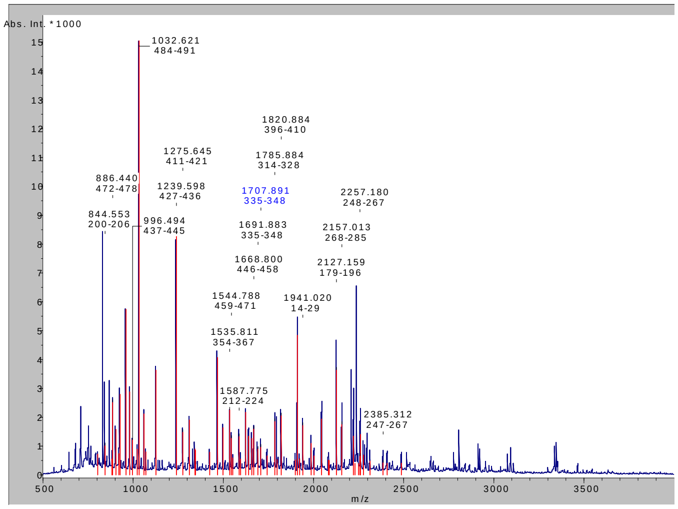

HQLYIDETVNSNIPTNLR, MS/MS ion score = 55

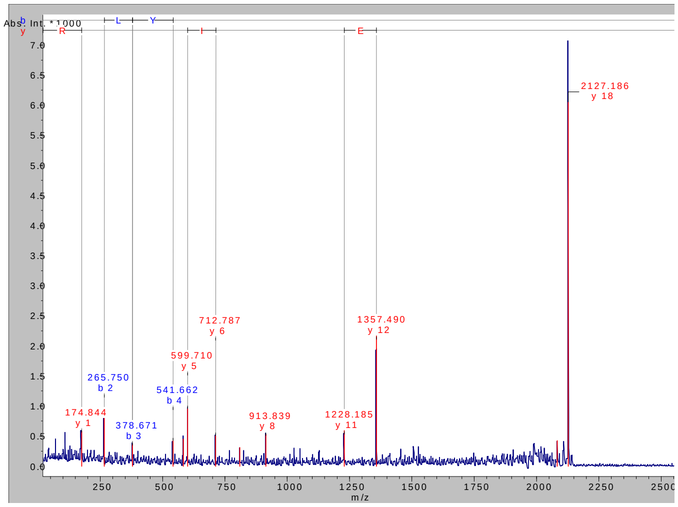

VYCDMNTENGGWTVIQNR, MS/MS ion score = 30

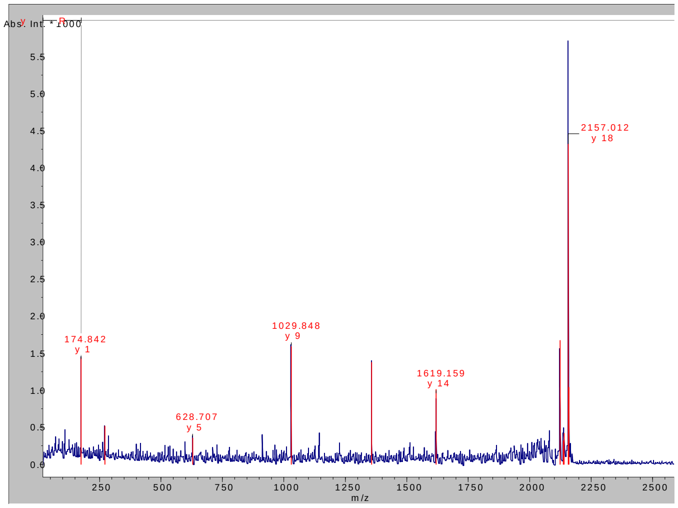

Spot IV: Haptoglobin (P00738)
PMF score = 112

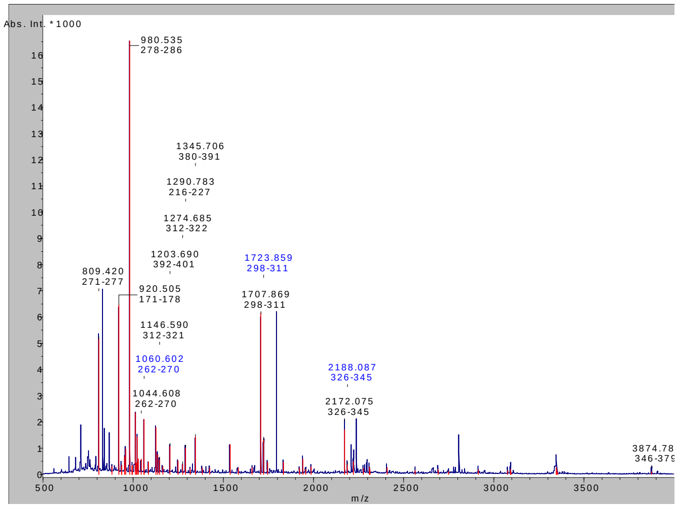

YVMLPVADQDQCIR, MS/MS ion score = 67

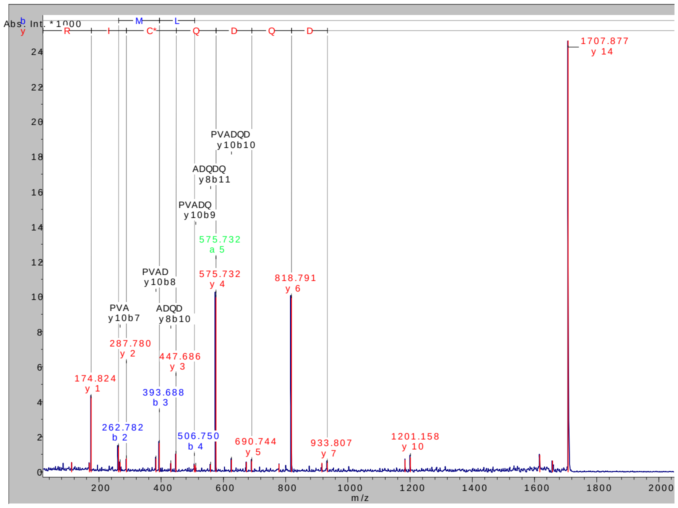

GSFPWQAK, MS/MS ion score = 33

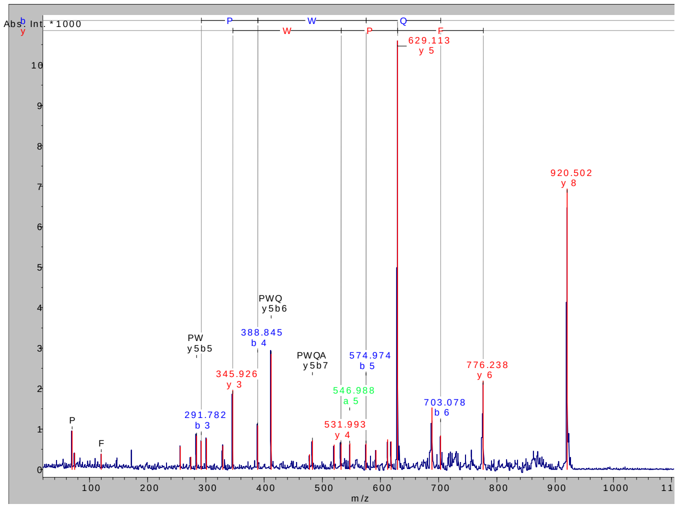

VGYVSGWGR, MS/MS ion score = 26

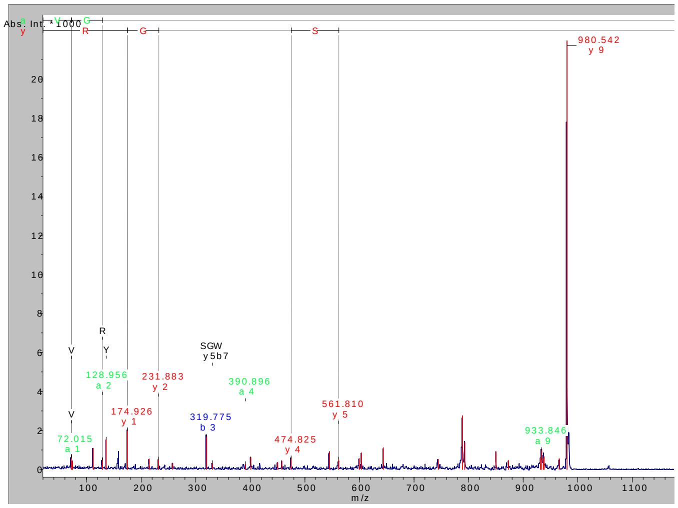

Spot V: Apolipoprotein E (P02649)

PMF score = 160

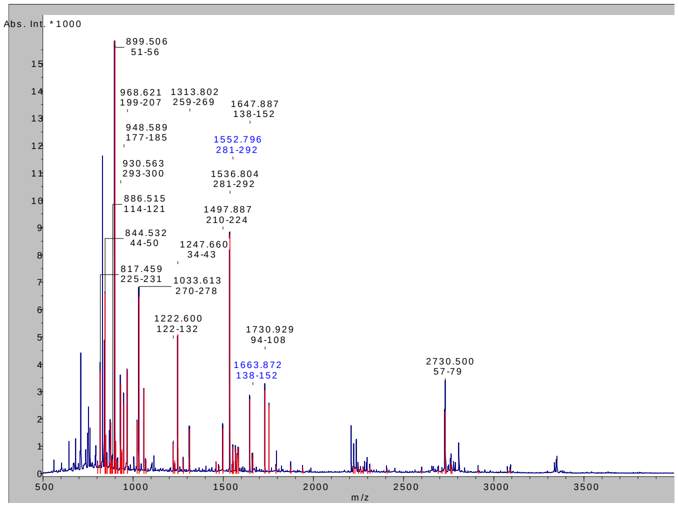

SWFEPLVEDMQR, MS/MS ion score = 53

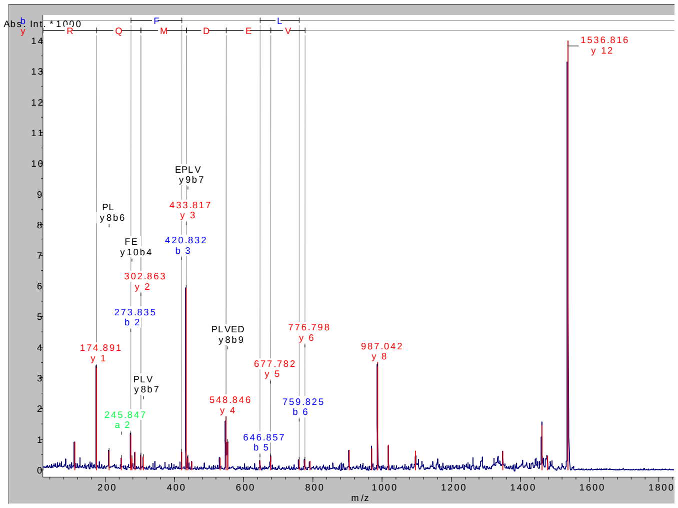

QQTEWQSGQR, MS/MS ion score = 28

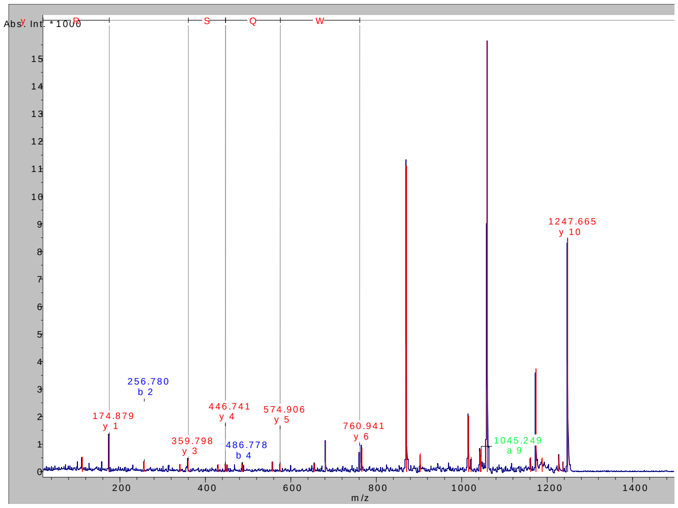

LQAEAFQAR, MS/MS ion score = 28

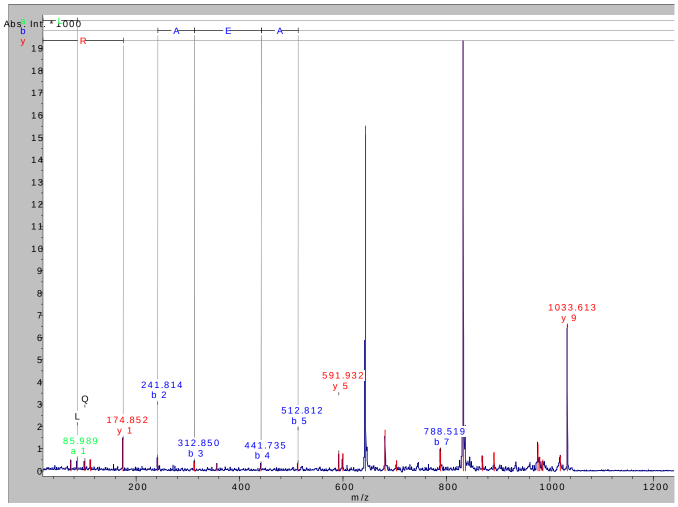

FWDYLR, MS/MS ion score = 14

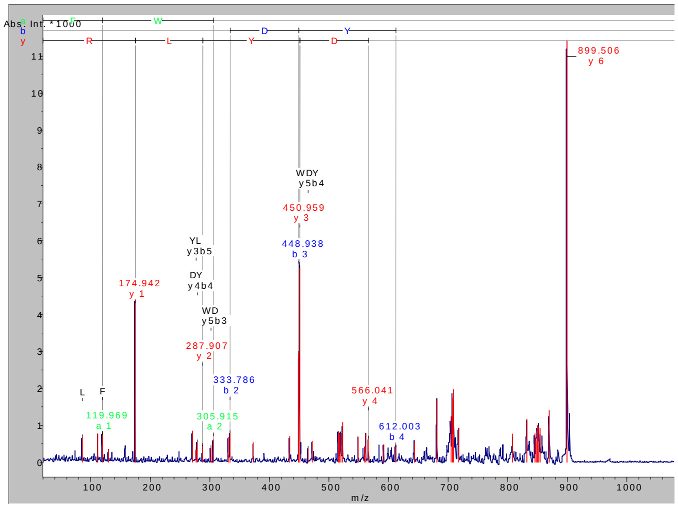

Spot VI: Prostaglandin-H2 D-isomerase (P41222)

PMF score = 63

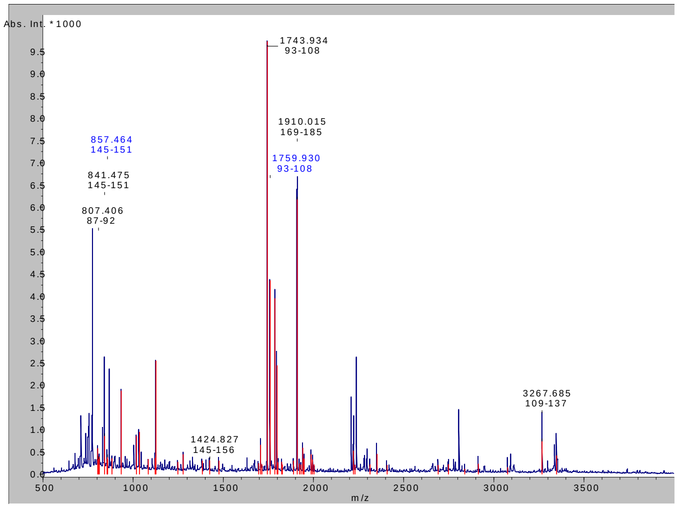

AQGFTEDTIVFLPQTDK, MS/MS ion score = 91

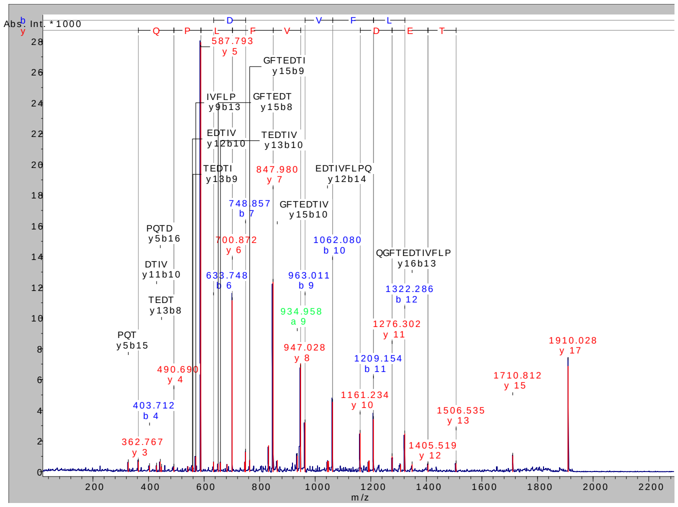

TMLLQPAGSLGSYSYR, MS/MS ion score = 70

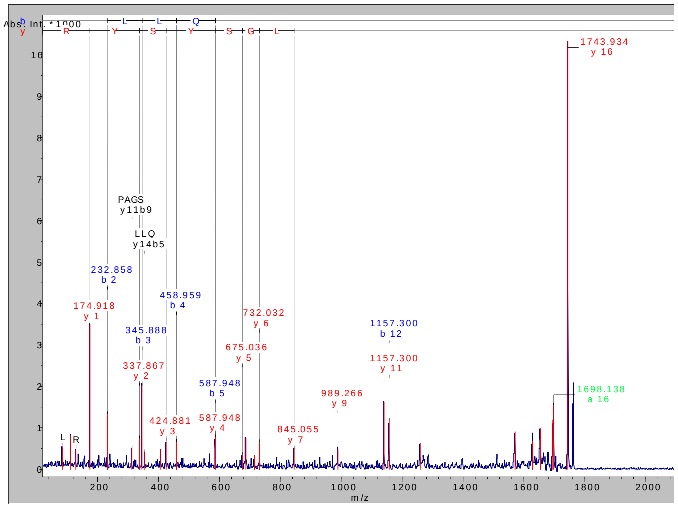

Supplement: Figure S3 — MS-spectra (0.21 MB PDF) [file pone.0010079.s003.pdf]
