# Supplementary material for: A Proteomic Approach for the Diagnosis of Bacterial Meningitis
Source: PLoS One. 2010 Apr 8;5(4):e10079. doi: 10.1371/journal.pone.0010079 (PMC2851643; doi:10.1371/journal.pone.0010079)
Supplement: Table S1 — Additional data of protein identification. Columns 4–6 refer to PMF searches; columns 7–9 refer to MS/MS ion searches. (0.05 MB DOC) [file pone.0010079.s004.doc]

91

70

AQGFTEDTIVFLPQTDK

TMLLQPAGSLGSYSYR

2

8

(44)

63

42

P41222

Prostaglandin-H2 D-isomerase

VI

53

28

28

14

SWFEPLVEDMQR

QQTEWQSGQR

LQAEAFQAR

FWDYLR

4

18

(48)

160

55

P02649

Apolipoprotein E

V

67

33

26

YVMLPVADQDQCIR

GSFPWQAK

VGYVSGWGR

3

15

(44)

112

35

P00738

Haptoglobin

IV

55

30

HQLYIDETVNSNIPTNLR

VYCDMNTENGGWTVIQNR

2

19

(40)

170

45

P02675

Fibrinogen beta chain

III

59

54

48

LADVYQAELR

QLQSLTCDLESLR

ITIPVQTFSNLQIR

3

28

(57)

184

64

P14136

Glial fibrillary acidic protein

II

29

12

CCHCCLLGR

TGYYFDGISR

2

7

(13)

82

9^c^

P23142

Fibulin-1

I

MS/MS ion score^b^

Peptide sequence

Number unique peptides

sequenced

Number masses

matched

(not matched)

PMF score^a^

PMF coverage [%]

Swiss-Prot accession

Protein name

Spot

No.

**Table S1: Additional data of protein identification.**

Columns 4-6 refer to PMF searches; columns 7-9 refer to MS/MS ion searches.

^a)^ Mascot protein score obtained for the peptide mass fingerprint (PMF). The significance threshold was 56.

^b)^ Mascot MS/MS ion scores obtained for the individual peptides sequenced. The significance threshold for identity was

25-30 depending on how many peptides fell within the mass tolerance window about the precursor mass.

^c)^ Fibulin-1 did not fulfill the stringent criteria with regard to PMF sequence coverage (>20%), but was considered

identified on the basis of the validation by 2D-Immunoblotting.
